# Retraction: MicroRNA-1271 modulates hepatitis B virus replication, cell proliferation and apoptosis in hepatitis B virus-related hepatocellular carcinoma by targeting SIRT1

**DOI:** 10.1039/d1ra90060a

**Published:** 2021-02-01

**Authors:** Laura Fisher

**Affiliations:** Royal Society of Chemistry Thomas Graham House, Science Park, Milton Road Cambridge CB4 0WF UK advances-rsc@rsc.org

## Abstract

Retraction of ‘MicroRNA-1271 modulates hepatitis B virus replication, cell proliferation and apoptosis in hepatitis B virus-related hepatocellular carcinoma by targeting SIRT1’ by Fei Tang *et al.*, *RSC Adv.*, 2019, **9**, 39904–39913, DOI: 10.1039/C9RA08248D.

The Royal Society of Chemistry hereby wholly retracts this *RSC Advances* article due to concerns with the reliability of the data. The images in the article, and the raw data provided by the authors, were screened by an image integrity specialist.

The blots and many other features of the article were found to closely resemble the blots and features of a number of other articles, which is highly unexpected given that there are completely different author lists for these articles. The western blots in these papers have very similar-looking blots with non-distinctive shaped bands at the same, regular distance throughout the articles. They do not look genuine.

In addition, analysis of the raw data provided by the authors for this paper showed that it was digitally cropped without molecular weight markers or any labelling on it. The raw data also shared striking similarities to the raw data provided for a number of other articles with no overlapping authors. The raw data does not look genuine and therefore cannot be used to validate the published data.

Given the significance of the concerns about the validity of both the data in the article and the raw data provided by the authors, the findings presented in this article are not reliable.

Fengmei Wang does not agree to the retraction. The other authors have been informed but have not responded to any correspondence regarding the retraction.

Signed: Laura Fisher, Executive Editor, *RSC Advances*

Date: 19^th^ January 2021

## Supplementary Material

